# Bispecific antibodies and dual-targeting CAR-T cells for multiple myeloma: latest updates from the 2023 ASCO annual meeting

**DOI:** 10.1186/s40164-023-00436-9

**Published:** 2023-08-26

**Authors:** Jiangxue Hou, Yufu Li, Quande Lin

**Affiliations:** The Affiliated Cancer Hospital of Zhengzhou University & Henan Cancer Hospital, Zhengzhou, 450008 China

**Keywords:** Multiple myeloma, Bispecific antibody, CAR T, Clinical trial

## Abstract

Bispecific antibodies (BsAbs) and dual-targeted chimeric antigen receptor T (CAR T) cells have been employed in relapsed/refractory multiple myeloma (RRMM) patients over the past few years, as an increasing number of patients were ineffectively treated by ≥ 3 prior lines of therapy. BCMA/CD3 and GPRC5D/CD3 are the most popular combinations. Clinical findings indicated that patients exhibit a greater susceptibility to stronger and more enduring responses. Here, we summarize the latest data from the 2023 ASCO annual meeting on BsAbs targeting BCMA/CD3, GPRC5D/CD3 and BCMA/CD19 CAR T cells.

**Supplementary Information:**

The online version contains supplementary material available at 10.1186/s40164-023-00436-9.

## To the editor

Although the outcome of relapsed/refractory multiple myeloma (RRMM) has significantly improved in recent years, a large proportion of patients treated with immunotherapy relapse [1]. However, bispecific antibodies (BsAbs) and dual-targeting chimeric antigen receptor T (CAR T) cells have shown good efficacy and safety in clinical scenarios [2] and can provide strong and durable responses [3]. This review summarizes the latest updates from the 2023 ASCO meeting.

## Bispecific antibodies

One poster reported the efficacy of the commercial drug teclistamab, the first BCMA/CD3 BsAb that received FDA approval for the treatment of RRMM. All patients were triple class refractory (TCR), 80% were penta-drug refractory, and ten had prior anti-BCMA therapy. The overall response rate (ORR) was 60% (9/15), and Grade 1–2 cytokine release syndrome (CRS) was observed in 41% of patients [4].

Elranatamab is another BCMA/CD3 BsAb. It had the longest median follow-up (15 months) among all phase 2 trials of this type of BsAb. In total, 123 patients received elranatamab therapy. Of these, 96.7% and 42.3% of patients were triple class and penta-drug refractory, respectively. The ORR (objective response rate) was 61%, and the duration of response (DOR) at 12 months (mo) was 74.1% [5].

The efficacy of linvoseltamab, a BCMA/CD3 BsAb, was demonstrated in an oral abstract. In two cohorts (200 mg vs. 50 mg), the ORR was 64% and 50%, while the incidence of Grade 1–2 CRS was 36% and 51%, respectively [6].

A phase I trial assessing the safety and preliminary efficacy of F182112, a BCMA/CD3 BsAb, was presented at this meeting. Sixteen patients were enrolled in this clinical study. The ORR was 43.8%, and the most common treatment-related adverse events (AEs) were CRS (81%), which were all Grade 1–2 [7].

The MonumenTAL-1 trial focused on investigating the efficacy and safety of talquetamab, a GPRC5D/CD3 BsAb. The ORR was 74%, 73% and 63% in the 0.4 mg/kg QW, 0.8 mg/kg Q2W, and other dose (some patients with prior T-cell redirection therapy received either dose) cohorts, respectively. The median progression-free survival (mPFS) was 7.5, 11.9 (61% censored), and 5.1 mo, respectively. The AEs were all clinically manageable [8].

The first results from the RedirecTT-1 study were presented at the 2023 ASCO meeting. This study aimed to investigate the outcome of the combination of two BsAbs, teclistamab (tec) targeting BCMA/CD3 and talquetamab (tal) targeting GPRC5D/CD3, simultaneously. The ORRs among evaluable patients and evaluable patients with extramedullary disease (EMD) were 84% (52/62) and 73% (19/26), the rates of CR or better (≥ CR) were 34% (21/62) and 31% (8/26), respectively. The ORR was 92% (12/13) in evaluable patients and 83% (5/6) in evaluable patients with EMD at the recommended phase 2 regimen (RP2R). The rate of CR or better (≥ CR) was 31% (4/13) and 33% (2/6), respectively, and AEs were manageable [9].

Another combination treatment was tal + daratumumab in the TRIMM-2 study. The ORR was 78% and it showed strong and durable responses with an mPFS of 19.4 mo in heavily pretreated RRMM patients. The 12-mo PFS and overall survival (OS) rates were 76% and 93%, respectively. In addition, the safety profile was acceptable [10].

In addition to all of the above trials, Shaji Kumar is conducting research to investigate the efficacy and safety of cevostamab, which targeted FcRH5 and CD3, in TCR RRMM patients in the CAMMA 2 study [11]. Relevant patient recruitment is currently in progress, although no empirical data have been obtained at this stage.

## Dual-targeting CAR-T cells

Previous results demonstrated that GC012F treatment led to a strong and durable response in RRMM patients. The same team provided updated trial data at the 2023 ASCO meeting (Abstract No. 8005). At the time of data cutoff, the ORR was 93.1%, and the rates of stringent complete response (sCR) and very good partial response (VGPR) were 82.8% (24/29) and 89.7% (26/29), respectively. All patients (29/29) achieved minimal residual disease (MRD) negativity (flow cytometry at 10^− 4^-10^− 6^), and the MRD-sCR rate was 82.8% (24/29) across all dose levels. The mDOR was 37.0 mo (95% CI, 11.0-NR), and the mPFS was 38.0 mo (95% CI, 11.8-NR). This encouraging outcome demonstrated that GC012F can further increase the clinical benefit to RRMM patients [12].

### Electronic supplementary material

Below is the link to the electronic supplementary material.

**Additional file 1**: Table 1. Properties of bispecific antibodies for multiple myeloma. Table 2. Outcomes of clinical trials of single-agent bispecific antibodies or combination therapy.

## Data Availability

The material supporting the conclusion of this study has been included within the article.

## Abbreviations

CAR: Chimeric antigen receptor
RRMM: Relapsed/refractory multiple myeloma
TCR: Triple-class refractory
BsAb: Bispecific antibody
GPRC5D: G protein-coupled receptor, family C, group 5, member D
ORR: Overall response rate/objective response rate
RP2R: Recommended phase 2 regimen
AEs: Adverse events
CRS: Cytokine release syndrome
DOR: Duration of response
EMD: Extramedullary disease
sCR: Stringent complete response
FcRH5: Fc receptor-homolog 5
