# Correction to: Bispecific antibodies and dual-targeting CAR-T cells for multiple myeloma: latest updates from the 2023 ASCO annual meeting

**DOI:** 10.1186/s40164-023-00447-6

**Published:** 2023-10-03

**Authors:** Jiangxue Hou, Yufu Li, Quande Lin

**Affiliations:** The Affiliated Cancer Hospital of Zhengzhou University & Henan Cancer Hospital, 450008 Zhengzhou, China

**Experimental Hematology & Oncology (2023) 12:74**

10.1186/s40164-023-00436-9

After online publication of the article1, the authors noticed Table 1 and 2 should have been published in the main article were inadvertently submitted and processed as Supplementary files.

The correct tables are published with this erratum.

**Table 1:** Properties of bispecific antibodies for multiple myeloma

Author	NCT No.	Agents	Ig type	Structural format	Target	Phase	References
Firestone R	N/A	Teclistamab	IgG4	1 + 1 symmetric	BCMA/CD3	post-marketing revaluation	[4]
Mohty M	NCT 04649359	Elranatamab	IgG2a	1 + 1 symmetric	BCMA/CD3	II	[5]
Lee HC	NCT03761108	Linvoseltamab	IgG4κ	1 + 1 symmetric	BCMA/CD3	I/II	[6]
Sun MY	NCT 04984434	F182112	--	--	BCMA/CD3	I	[7]
Morillo D	NCT 03399799	Talquetamab	IgG4PAA	1 + 1 symmetric	GPRC5D/CD3	I	[8]
Schinke CD	NCT 04634552	Talquetamab	IgG4PAA	1 + 1 symmetric	GPRC5D/CD3	I/II	[8]
Bachier CR	NCT05535244	Cevostamab	IgG1	1 + 1 symmetric	FcRH5/CD3	I/II	[11]

Abbreviations: FcRH5: Fc receptor-homolog 5; GPRC5D: G protein-coupled receptor, family C, group 5, member D; IgG4PAA : immunoglobulin G4 proline, alanine, alanine

**Table 2:** Outcomes of clinical trials of single-agent bispecific antibodies or combination therapy

NCT No.	N/A	NCT4649359	NCT03761108	NCT4984434	NCT03399799	NCT04586426	NCT04108195
					NCT04634552		
Target	BCMA/CD3	BCMA/CD3	BCMA/CD3	BCMA/CD3	GPRC5D/CD3***	BCMA/CD3 + GPRC5D/CD3	GPRC5D/CD3 + CD38
Drug	Teclistamab	Elranatamab	Linvoseltamab	F182112	Talquetamab	Teclistamab + Talquetamab	Talquetamab + Daratumumab
Patient Number	24	123	179 (200 mg: n = 75 50 mg: n = 104)	16	143(QW) 145(Q2W) 51(prior T therapy)	63	65
Median age (year)	66 (51–80)	68 (36–89)	66** (37–90)	64 (52–74)	N/A	67 (39–81)	63 (37–81)
Median prior LOT	7 (4–13)	5 (2–22)	5** (1–16)	≥ 4 (56%)	5–6	5 (1–11)	5 (2–16)
TCR MM	100%	96.7%	81%**	N/A	74% 69% 84%	78%	58%
Median time to response(mo)	0.53	NR (95%CI, 12.9-NE)	N/A	N/A	N/A	N/A	1 (0.9–8.3)
ORR	60%	61%* (95%CI, 51.8–69.6)	64%(include 12 patients in Phase I) 50%	43.8% (95%CI, 19.8–70.1)	74% 73% 63%	84%	78%
≥CR	N/A	31.7%	N/A	N/A	(≥ VGPR)59% 57% 53%	34%	45%
Median follow-up(mo)	1.3	12.8 (0.2–22.7)	2.3 4.7	3.1 (0.9–11.7)	14.9 8.6 11.8	14.4 (0.5–21.9)	11.5 (1.0-27.3)
Median PFS(mo)	N/A	NR	N/A	N/A	7.5 11.9 5.1	N/A	19.4
Mediann OS(mo)	N/A	NR	N/A	N/A	N/A	N/A	N/A
12-mo PFS	N/A	57.1% (95%CI, 47.2%-65.9%)	N/A	N/A	N/A	N/A	76%
12-mo OS	N/A	62% (95%CI, 52.8%-70%)	N/A	N/A	N/A	N/A	93%
CRS/ICANs	Gr 1–2 CRS 41%	N/A	Gr1-2 CRS:36% 51% Gr ≥ 3 ICANS:2% 1%	Gr 1–2 CRS: 81%	Gr1-2 CRS:79% ICANS:11% 75% 11% 77% 3%	Gr 1–2 CRS 78%; Gr 3 CRS 3%; ICANS :1patient	Gr 1–2 CRS 78%; Gr 1–2 ICANS 5%
Infection	N/A	N/A	Gr1-2:17%;Gr ≥ 3:26% 28% 31%	N/A	58% 65% 71%	N/A	63%
References	[4]	[5]	[6]	[7]	[8]	[9]	[10]

Abbreviations: N/A: not applicable; NR:not reached; NE:not evaluated; LOT: line of therapy; TCR: triple-class refractory ;EMD: extramedullary disease; ORR: overall response rate; CR:complete remission; AEs: Adverse events Gr: Grade; CRS: cytokine release syndrome; ICANS: immune effector cell–associated neurotoxicity syndrome

*:objective response rate

**:when evalucated, 73 patients in Phase I were enrolled

***:In this trial, patients were separated into three cohorts, 143 patients received talquetamab 0.4 mg/kg QW, 145 were 0.8 mg/kg Q2W, 51 patients with prior T-cell redirection therapy received either dose

